# Shaping and Centering Ability, Cyclic Fatigue Resistance and Fractographic Analysis of Three Thermally Treated NiTi Endodontic Instrument Systems

**DOI:** 10.3390/ma13245823

**Published:** 2020-12-21

**Authors:** Saulius Drukteinis, Vytaute Peciuliene, Ruta Bendinskaite, Vilma Brukiene, Rasmute Maneliene, Vygandas Rutkunas

**Affiliations:** Institute of Dentistry, Faculty of Medicine, Vilnius University, Zalgirio 115, LT-08217 Vilnius, Lithuania; vytaute.peciuliene@mf.vu.lt (V.P.); ruta.bendinskaite@mf.vu.lt (R.B.); vilma.brukiene@mf.vu.lt (V.B.); rasmute.maneliene@mf.vu.lt (R.M.); vygandas.rutkunas@mf.vu.lt (V.R.)

**Keywords:** cyclic fatigue, fractographic analysis, micro-computed tomography, nickel–titanium, rotary movement, root canal shaping, thermal treatment, transportation

## Abstract

The better understanding of the clinically important behavioral features of new instrument systems has an important significance for the clinical endodontics. This study aimed to investigate the shaping and centering ability as well as cyclic fatigue resistance of HyFlex CM (CM), HyFlex EDM (EDM) and EdgeFile (EF) thermally treated nickel–titanium (NiTi) endodontic instrument systems. Sixty curved root canals of the mesial roots of mandibular molars were randomly assigned into three groups (*n* = 20) and shaped using CM, EDM and EF files up to the size 40 and taper 04 of the instruments. µCT scanning of the specimens before and after preparation was performed and the morphometric 2D and 3D parameters were evaluated in the apical, middle and coronal thirds of root canals. In each group, 40.04 instruments (*n* = 20) were subjected to the cyclic fatigue resistance test in artificial root canals at 37 °C temperature until fractures occurred, and the number of cycles to failure (NCF) was calculated. The fractographic analysis was performed using a scanning electron microscope, evaluating topographic features and surface profiles of the separated instruments. The one-way analysis of variance with post hoc Tuckey’s test was used for statistical analysis of the data; the significance level was set at 5%. All systems prepared the comparable percentage of root canal surface with the similar magnitude of canal transportation in all root thirds (*p* > 0.05), but demonstrated significantly different resistance to cyclic fatigue (*p* < 0.05). The most resistant to fracture was EF, followed by EDM and CM. The length of the fractured fragments was not significantly different between the groups, and fractographic analysis by SEM detected the typical topographic features of separated thermally treated NiTi instrument surfaces.

## 1. Introduction

Despite all the technological innovations in endodontic instruments and devices, the root canal instrumentation procedure is still challenging for the clinicians [1]. Nickel–Titanium (NiTi) endodontic instruments were launched to the market and clinical practice in 1988, significantly improving the quality of root canal shaping and reducing preparation mishaps and errors [2]. They increased the success of the outcome of primary root canal treatment in comparison with the non-flexible stainless steel instruments that were previously used for preparation [3]. This success is related to the better superelasticity of the instruments and their capability to preserve the original three-dimensional (3D) configuration and curvature of the root canal, with less transportation, zipping and ledging after preparation [4].

Despite demonstrated advantages, the instruments made from conventional NiTi alloy possess some undesirable properties—they have shape memory effect, which is related to the possibility of root canal transportation, and they are not completely resistant to deformation and fractures during the shaping procedures [3]. Moreover, due to the mechanical properties of the NiTi alloy, clinicians are unable to detect any deformations and defects on the working part of the NiTi instruments when used, preventing the possible separation of the instrument during root canal preparation [5]. Studies have demonstrated, that the fractures of NiTi instruments are directly related to the cyclic and torsional fatigue failures, and there is no possibility to avoid it clinically, as files are continuously affected by compressive and tensile stresses during root canal shaping [6,7].

To reduce or eliminate the undesirable shape memory effect of the conventional NiTi endodontic instruments, to enhance mechanical features and increase resistance to stresses and fatigue, the concept and technologies of thermal treatment of traditional NiTi alloy were introduced [8]. The thermal or thermomechanical treatment makes NiTi instruments much more deformable, pseudoplastic and with controlled memory effect [9]. The thermomechanically treated instruments do not straighten curved root canals during preparation and ensure less root canal transportation [7]. Additionally, it has been shown, that thermally treated NiTi endodontic instruments have significantly enhanced resistance to cyclic fatigue and fractures in comparison to conventional NiTi or M-wire NiTi instruments [10,11].

HyFlex CM (Coltene-Whaledent, Allstetten, Switzerland) is the rotary instrument system, made from a substantially new type of NiTi wire, subjected to the specific thermomechanical processing and named as a controlled memory (CM) wire [11]. The files with controlled memory are extremely flexible, but without the shape memory, which is typical for the conventional NiTi instruments [12]. HyFlex EDM (Coltene-Whaledent) and HyFlex CM (Coltene-Whaledent) instruments are made from the same CM-wire. However, HyFlex EDM instruments are the first instruments manufactured using a electrical discharge machining (EDM) process [13]. EDM is a non-contact machining procedure, precisely melting and evaporating the wire’s surface via pulsed electrical discharge [9]. The surface of the EDM processed files is harder, instruments are more resistant to the fractures, due to significantly enhanced resistance to cyclic and torsional fatigue [6]. EdgeFile (EdgeEndo, Albuquerque, NM, USA) endodontic instruments are made from Fire-Wire™ annealed heat-treated NiTi alloy, which increases flexibility, flexural strength and resistance to cyclic fatigue of the files [14]. The controlled memory wire, which is used for grounding of the instruments, ensures the better centering ability of the files during rotation and less root canal transportation in comparison to traditional NiTi alloys [15].

Micro-computed tomography (µCT) has been widely used for the two- and three-dimensional (2D, 3D) evaluation of shaped root canals due to the noninvasiveness, repeatability and high accuracy of the technique [7,11]. The assessment of cyclic fatigue resistance and subsequent fractographic analysis of the fractured fragments using scanning electron microscopy (SEM) is the most commonly used methods to evaluate the resistance to fractures and topographic profile of the surfaces of the separated instruments [13,14]. The published scientific data regarding the 3D (volume, area, unprepared surfaces) and 2D (canal transportation, the thickness of the remaining dentin) changes in root canal geometry as well as a cyclic fatigue resistance of thermally-treated instruments is still lacking and controversial [12,16]. Therefore, this study aimed to evaluate and compare the shaping and centering ability of three thermally treated NiTi instrument systems in a moderately curved canals of mandibular molars using µCT, and their resistance to cyclic fatigue. The null hypotheses tested were: (a) the instrument systems would demonstrate the similar shaping and centering ability, and (b) the files would be equally resistant to cyclic fatigue.

## 2. Materials and Methods

### 2.1. Assessement of the Shaping and Centering Ability

#### 2.1.1. Specimen Preparation

The approval of the ethical committee has been obtained for this investigation (protocol No. EK-2). Thirty mandibular molars were selected for the study. The inclusion criteria were: the mesial roots with a moderate 10°–20°curvature, according to Schneider’s criteria [17] and two separate root canals in the root. The endodontic access cavities were opened and shaped using size No.3 EndoAccess (Dentsply Sirona, Ballaigues, Switzerland) burs and the presence of two separate mesial canals in the root was established, inserting a size 10 K-file (Dentsply Sirona) to the working length (WL). The specimens were decoronated at the level of the cementoenamel junction using EndoAccess burs.

The WL was established with a size 10 K-file inserting the instrument into the canal until the tip was noticeable at the apical foramina, subsequently retracting the instrument 1 mm and confirming the WL with an endodontic ruler. The glide path was created using a size 15 and 20 K-Flexofile instruments and the teeth were randomly assigned into three experimental groups (*n* = 20). In each group, the root canals were prepared using CM, (EDM) or EdgeFile (EF) files and the crown-down technique up to the final files size 40 and 0.04 taper, according to the manufacturer’s instructions.

CM and EDM instruments were used following the manufacturer’s recommendations at the rotation speed of 500 rpm and torque control level of 2.5 N/cm using the 8:1 reduction handpiece powered by Genius^®^ endodontic motor (Ultradent Products Inc., South Jordan, UT, USA). To shape the root canals, the following set of the CM instruments was used: 25/0.08 file was used to prepare the orifices and coronal third, while the 20/0.04, 25/0.04, 30/0.04 and 40/0.04 instruments were used up to the full WL. In the EDM group canals were instrumented with 25/0.12 Orifice opener, following with 25/~ HyFlex OneFile and 40/0.04 Finishing File to the full WL. In EF group the instruments of the X5 series were used with the same endodontic motor at a rotational speed of 300 rpm and torque of 2.0 N/cm in the following sequence: 20/0.04, 20/0.06, 30/0.04, 30.06 and 40/0.04.

Root canals were irrigated with 5 mL solution of 3% NaOCl (Ultradent Products Inc.), while at the end of the cleaning-shaping procedure the 5 mL of 18% EDTA (Ultradent Products Inc.) for 2 min was used as a final flush. The disposable 5 mL syringes and 31-G NaviTip needles (Ultradent Products Inc.) were used for the delivery of the solutions. After preparation the root canals were dried with paper points, and the specimens were subjected to postoperative scanning. All cleaning and shaping procedures were performed by the experienced endodontist with the prior training on the use of the instrument systems.

#### 2.1.2. µCT Imaging and Analysis

The pre-and postoperative scanning of the specimens was performed using a high-resolution µCT scanner SkyScan 1173 (Bruker-microCT, Kontich, Belgium) and following settings: 10 kV, 50 mA and an isotropic voxel size of 22.8 μm, 1 mm aluminum filter, 180° rotation and a rotation step of 0.18. For the reconstruction of the images, the NRecon v.1.6.9 (Bruker microCT) software was used with the settings of the ring artefact reduction factor of 5 and beam hardening correction of 30%.

The 9 mm of each mesial root (from the tip to the cementoenamel junction) were selected as the volume of interest (VOI) for morphometric 3D analysis. The pre- and post-preparation cross-sectional images were segmented, superimposed and co-registered using the DataViewer v.1.5.1 software (Bruker microCT). The scanning data were analyzed using CTAn v.1.14.4 software (Bruker microCT) and visualized using CTVol 1.10.1.0 software (Bruker microCT).

**Figure 1:**
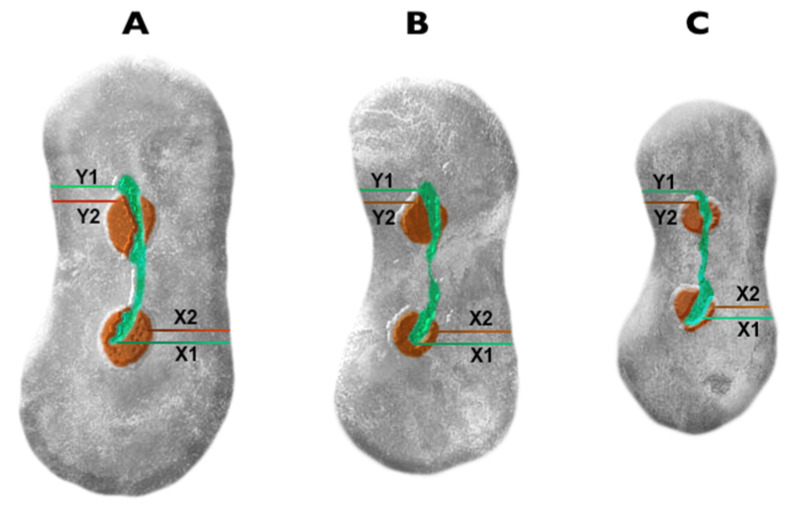
Cross-sectional images representing reference points of measurements before (greenish) and after (reddish) root canal shaping at the level of 9 (**A**), 6 (**B**) and 3 mm (**C**) from the apex subsequently. X1 represents the shortest distance from the mesial edge and the Y1 from the distal edge of the unprepared root canal to the appropriate edge of the root. X2 and Y2 represent the shortest distance from the mesial and distal edge of the prepared root canal to the relevant edge of the root [19].

The amount of the root canal transportation was evaluated using co-registered cross-sectional images, measuring the dentine thickness of the mesial and distal root walls in millimeters (Figure 1). Subsequently, the obtained values were applied to the formula proposed by Gambill et al. [18]. All measurements were done at the coronal, middle and apical thirds corresponding to the level of 9, 6, 3 mm from the root apex respectively. According to the formula, the value equivalent to 0.0 indicates no canal transportation.

### 2.2. Evaluation of the Cyclic Fatigue Resistance

**Figure 2:**
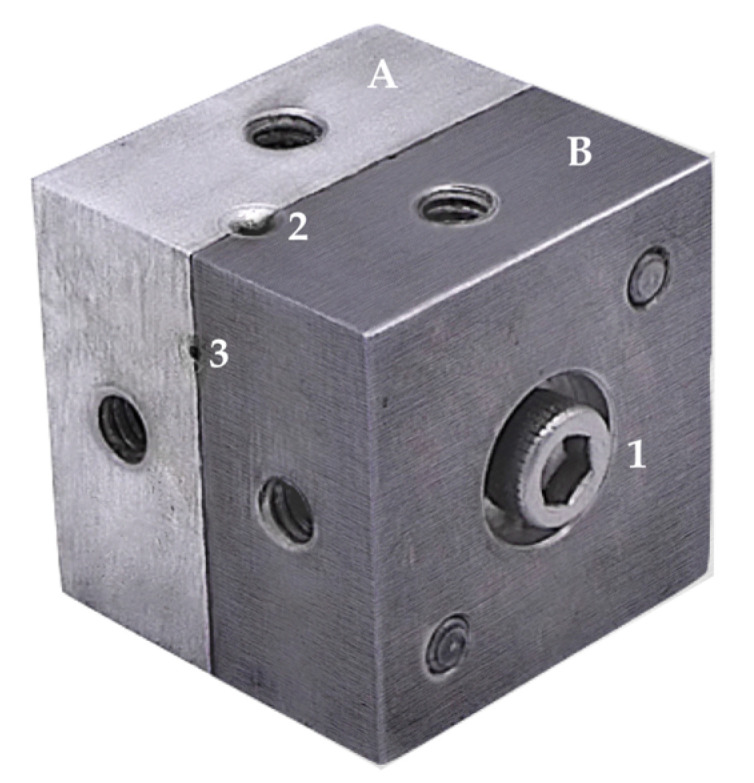
Two adjustable metal blocks (**A**,**B**) were screwed (1) to make a cyclic fatigue testing device. The tested instrument was inserted into the simulated canal via “orifice” (2) up to the terminal point (3) and subjected to rotation [19].

Twenty new CM, EDM and EF instruments (40/0.04/25 mm) were subjected for to cyclic fatigue testing. The metallic cyclic fatigue testing device with an artificial canal of 60° angle and 5.0 mm radius of curvature was produced for the experiment as described previously (Figure 2) [19]. Before the cyclic fatigue test, the customized metallic device was submerged into the thermal bath (Thermo Scientific™ Precision™; Fisher Scientific; Vantaa, Finland) containing 37 °C water, to simulate body temperature.

The instruments were inserted into a simulated canal and subjected to the rotary movement until their failure and fractures. The CM and EDM files were used at the speed of 500 rpm, while EF instruments were rotated at the speed of 300 rpm, according to manufacturer’s recommendations. The time to fracture in seconds was recorded visually by the operator under the ×2.4 magnification (MO Optics^®^ Ultralight Flip-up loupes; MeridentOptergo AB, Mölnlycke, Sweden) in seconds using the digital desktop stopwatch. The number of cycles to failure (NCF) was calculated using the widely accepted formula: NCF = rotation time to failure (seconds) × rotation speed/60. The length of the separated instrument fragments was calculated with a digital micro caliper (Mitutoyo, Kawasaki, Japan).

### 2.3. Fractographic Analysis

Two new and three fractured NiTi files of each instrument system were attached on aluminum holders and observed using SEM (T3030; Hitachi Ltd., Tokyo, Japan). The fractographic features of the external surfaces, tips and fractured cross-sections were assessed using a secondary electron image mode at 15 kV and ×50, ×200, ×1000, ×3000 magnifications.

### 2.4. Statistical Analysis

The data of the morphometric 2D and 3D parameters were submitted to the Shapiro-Wilk test, which demonstrated the normal distribution. The one-way analysis of variance with post hoc Tuckey’s test was used for comparisons between the groups; the significance level was set at 5%. The SPSS 25.0 software (SPSS Inc., Chicago, IL, USA) was used for data analysis.

## 3. Results

### 3.1. Shaping and Centering Ability

There were no differences in the preoperative volume of the root canals before shaping between the three groups (*p* > 0.05), indicating the equal initial parameters of the samples. Therefore, the morphometric parameters between groups after instrumentation also did not differ significantly (Table 1). All instrument systems were equally effective in shaping ability and demonstrated comparable volume of removed dentin and the percentage of unprepared root canal wall surfaces (*p* > 0.05).

CM, EDM and EF rotary file systems displayed no significant differences in terms of centering ability and the magnitude of root canal transportation (*p* > 0.05) (Table 2).

**Figure 3:**
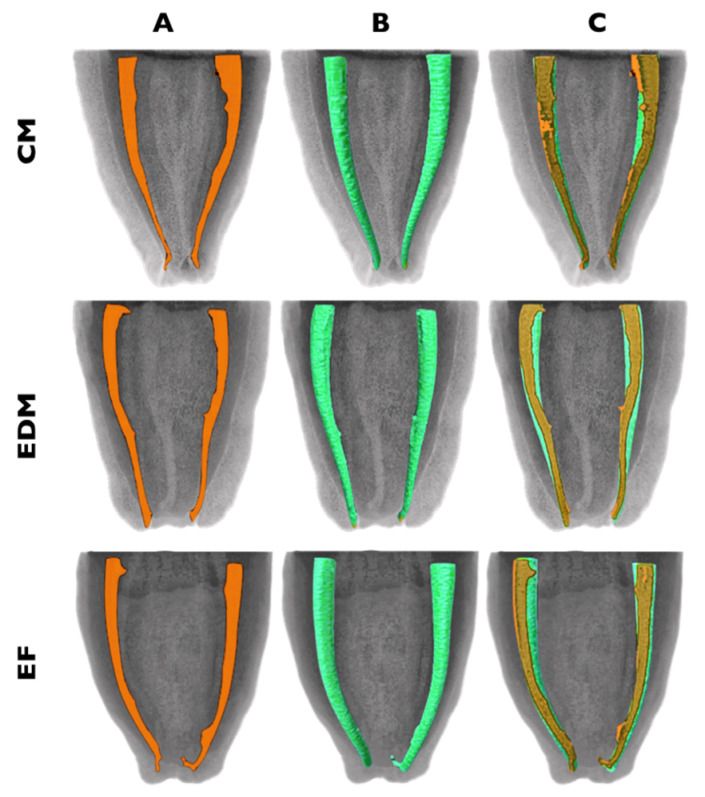
3D illustrative examples of the shaping ability of the thermally treated instrument systems: preoperative (**A**), postoperative (**B**) and superimposed reconstructions (**C**).

Overall, the CM, EDM and EF rotary files shaped root canals with no substantial preparation mishaps or errors and maintained the original root canal curvature with minimal transportation. The descriptive images of the 3D reconstructions of the mesial roots of the samples are shown in Figure 3.

### 3.2. Cyclic Fatigue Resistance Test

The range of the instrument rotation time before fracture varied significantly (Table 3). The average time to fracture for CM instruments was 136 s, for EDM files −376 s and EF −672 s. Hereby the EF exhibited the highest number of cycles to fracture (NCF), followed by EDM and CM instruments, while the CM files were the least resistant to cyclic fatigue. These NCF values were statistically significant between the experimental groups (*p* < 0.05). No statistically significant differences in the length of the fractured fragments were detected between three instrument groups (*p* > 0.05).

### 3.3. Fractographic Analysis

**Figure 4:**
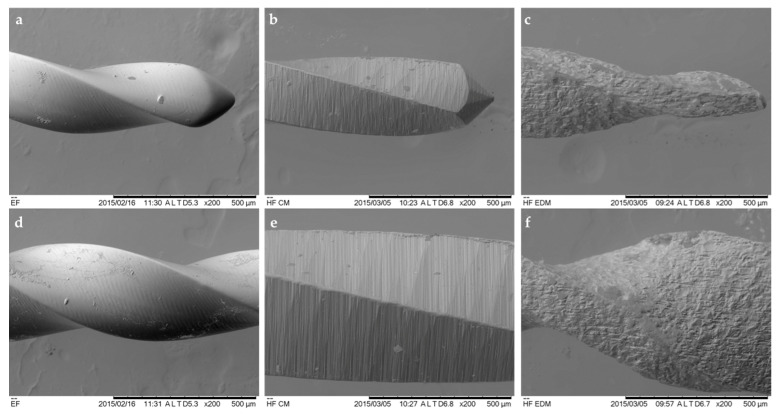
Micrographs of new thermally treated EF (**a**,**d**), CM (**b**,**e**) and EDM (**c**,**f**) endodontic instruments, demonstrating significant topographic differences of the surfaces (magnification ×200).

The superficial observations by SEM of the new CM, EDM and EF instruments revealed some substantial differences between the three instruments, indicating the different nature of the manufacturing process (Figure 4). The surface and the tip of the EG file was considerably smoother, and uniform in comparison to CM and EDM instruments. Multiple milling marks and scratches perpendicular to the long axis of the file were detected on the surface of the flutes and the tip of the CM instruments. Therefore, the typical crater-like irregular surface texture of the EDM files was detected. The nonuniform profile of the instrument was derived from the ED-machined manufacturing process.

**Figure 5:**
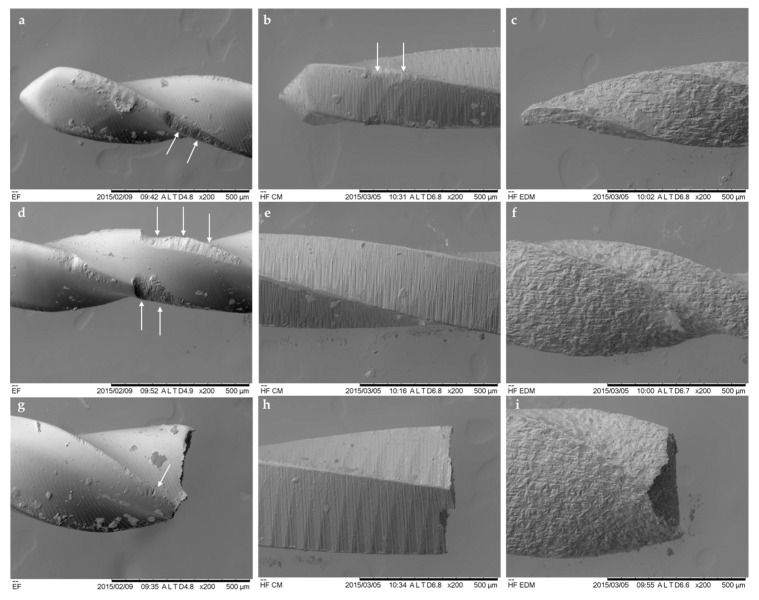
The SEM analysis under ×200 magnification revealed significant differences of the integrity of the instrument surfaces after cyclic fatigue test. The microscopic defects (white rows) were extensively evident on EF instruments (**a**,**d**,**g**); the CM files demonstrated minimal distortions (**b**,**e**,**h**), while the EDM instruments did not demonstrate any visible defects (**c**,**f**,**i**).

The fractographic analysis of the used and fractured instruments has shown differences between the appearance of the surfaces of the three instruments (Figure 5). The multiple microcracks and metal defects were visible on the EF instruments in conjunction with disrupted integrity of the blades and the tip. Meanwhile, the CM instruments demonstrated minor superficial defects on the flutes; however, the outline of the files was not significantly altered. The EDM files did not demonstrate any microscopic signs of distortion of the surfaces of the working part of the files. No blade disruption or microcracks were detected, indicating that even after numerous cycles of rotation, the instruments preserve the integrity of the crater-like irregular surface.

**Figure 6:**
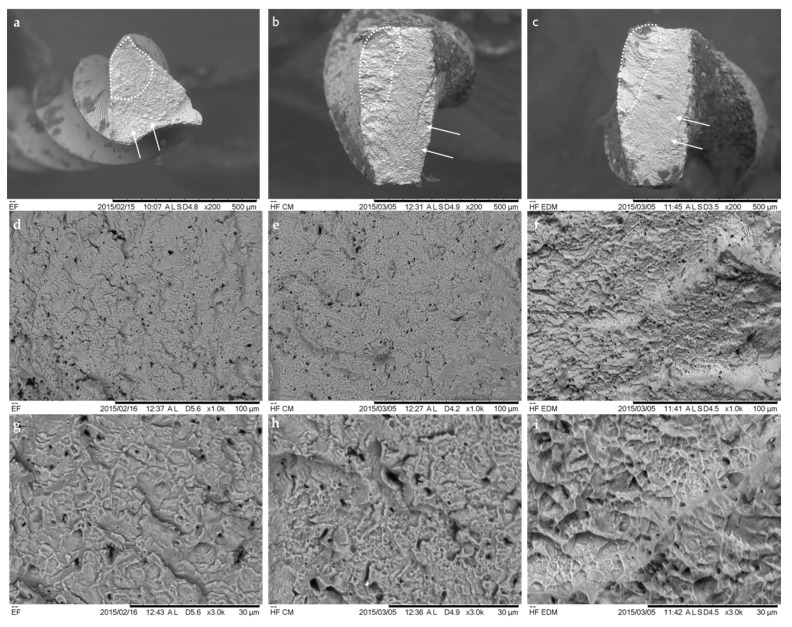
Representative SEM micrographs of the cross-sectional surfaces of fractured files at the ×500 (upper row), ×1000 (middle row) and ×3000 (lower row) magnifications. The typical fatigue propagation zone (dotted line) and dimpled area (white rows) were observed on EF (**a**), CM (**b**) and EDM (**c**) instruments. On higher magnifications, the detailed features of the dimpled area of EF (**d**,**g**), CM (**e**,**h**) and EDM (**f**,**i**) thermally treated files are evident.

The cross-sectional SEM observation at the lower magnification of the fatigued files revealed typical topographic features of the fractured NiTi instruments (Figure 6). The multiple crack origins at the crack initiation and propagation areas with the striation zones were visible, especially on the EF files. Meanwhile, the CM and EDM instruments exhibited wider dimpled areas in comparison to EF instruments. At high magnification (×1000 and ×3000) the ultrastructure of the surfaces were characterized as a rough, crater-like surface, with multiple microscopic dimples, pores and irregularities, especially on the EDM file surfaces.

## 4. Discussion

The present study evaluated the wide variety of clinically important features and properties of three thermally treated rotary NiTi instrument systems. There were no significant differences between systems in postoperative morphological 3D and 2D parameters of the shaped root canals; therefore, the first null hypothesis was accepted. However, the second null hypothesis was rejected as the resistance of the instruments to fractures under the torsional stresses differed significantly.

The assessment and comparison of the shaping ability of CM, EDM and EF instruments were tested on the moderately curved mesial canals of the extracted first mandibular molars, which are the most commonly endodontically treated teeth and represents the majority of the complicated features of internal anatomies, such as narrow and irregular shape, isthmuses and V-zones, significant apical curvature and, etc. [20,21]. On the other hand, due to the complexity and differences in the internal root canal anatomy, the selection of the equal samples is always challenging when natural teeth are used [22]. In order to homogenize the sample size parameters such as the length, initial root canal volume, anatomy and the curvature of the roots were included in this study. The microCT imaging, as the method used for the observations of the 3D and 2D morphometric parameters was selected due to the accuracy quantifying and qualifying these values of the different instrument systems [7,23].

The investigated CM, EDM and EF instrument systems differed in their flutes and cross-sectional design, the method of the thermal treatment of the instrument alloy, rotation speed and torque and the number of the files in the clinically used sequences. The size and the taper of the last instrument used for the root canal preparation was identical in all experimental groups (40/0.04), therefore excluding the possible negative impact of different sizes and tapers of the used instruments on the postoperative 3D values. Previous investigations have shown that thermally treated file systems are not superior in their shaping abilities in comparison to each other or conventional and M-wire NiTi instruments, despite their design and kinematics [7,24,25]. In this study, comparison between groups did not determine any significant differences regarding the changes in morphological 3D parameters after root canals shaping for all thermally treated file systems, and these results are in agreement with previous findings [26,27]. The previous study has found that the uninstrumented surface in curved root canals for HyFlex CM instruments was 41.26%, and these findings are in concordance with our results [11]. Overall, the relatively high percentages of untouched root canal walls by instrumentation, which can reach 47% or even more, depend on the internal anatomy and geometry of the root canals, instruments design, size, taper, kinematics, instrumentation technique or even skills of the operator [1,11,28,29]. Therefore, the copious root canal irrigation with agitation of these irrigants were recommended to improve the debridement and cleanliness of the root canal system after chemomechanical preparation [30,31].

Wu et al. [32] have shown that the apical root canal transportation can compromise cleanliness, disinfection and sealability of the root canals. It has been confirmed that transportation exceeding 0.3 mm can negatively impact the long term prognosis of endodontic treatment, while transportation up to 0.15 can be acceptable [1]. The numerous studies demonstrated that thermally treated rotary or reciprocating NiTi endodontic instruments possess the better centering ability and produce less root canal transportation in comparison to the conventional or even M-wire NiTi endodontic instruments [21,33]. Our results are in agreement with these findings, demonstrating the minimal changes in postoperative 2D morphological parameters and root canal transportation by rotary NiTi endodontic instruments with different design and thermal treatment approaches [15,21,24]. In this study, the magnitude of root canal transportation for CM, EDM and EF instruments did not even reach the acceptable transportation level of 0.15 mm; in contrast, the amount of the transportation was more than twice less the acceptable values. It indicates that all three instrument systems can ensure well centered preparation of the moderately curved root canals. Therefore, the possible clinical relevance and impact of the findings on the treatment outcome are questionable.

The design and cinematics of the files, as well as the properties of the alloy used for manufacturing of the instruments have a significant impact on cyclic fatigue resistance of the endodontic instruments [10]. Additionally, the anatomical factors of the root canals, such as geometry and curvature, play a substantial role too. However, it has been demonstrated that for the comparative analysis of the cyclic fatigue resistance the use of the natural teeth is problematic due to difficulties in selecting the identical degree and radius of curvature for comparative investigations. Therefore, the standardized severely curved artificial root canals were selected, as recommended previously [19]. Moreover, in this study the length of the fractured fragments of all file systems evaluated did not differ, indicating the correct position of the investigated files in the simulated canals during rotation and applying the comparable tensile and compression stresses on the instruments [34,35]. It has been shown that the environmental temperature in which NiTi files are tested for cyclic fatigue resistance has a crucial significance for the results [36,37]. Therefore, in this study, the customized metal block was submerged in 37 °C water, to simulate the body temperature and real clinical conditions. According to the results of this study, the EF instruments demonstrated significantly higher resistance to cyclic fatigue in comparison to EDM and CM instruments. These differences can be related to the different thermal treatment approaches of alloys of the instruments subjected to the fatigue test. Previous investigations are in agreement with the findings, demonstrating superior or even exceptional resistance of the Fire-Wire™ NiTi alloy EdgeFiles to cyclic fatigue in comparison to other thermally treated or conventional NiTi instruments at the 37 °C testing temperature [37,38]. In this investigation, the EDM files demonstrated better resistance to cyclic fatigue in comparison with CM instruments, and these results are in concordance with previous findings [39]. It was concluded that the higher resistance of EDM instruments is related to the thermomechanical treatment of the files, making the surface of the instrument harder and the file more resistant to stresses during rotation [13,40]. Moreover, it could be argued that the different rotation speed of the instruments potentially had an impact on the results of our investigation. However, it has been demonstrated, that rotation speed does not significantly affect the fatigue life of NiTi rotary instruments [41].

For the fractographic analysis of the instruments before and after fractures, the SEM in secondary electrons mode was used as proposed by previous studies [13]. The assessment revealed different topographic characteristics of three files, which can be mainly related to the different manufacturing techniques applied to the EF, CM and EDM instruments. It has been well documented that polishing and smoothening of the file surface reduce the amount of superficial defects and increase the resistance of the instrument to the fractures [9]. The results of this study confirm these findings, as the SEM investigation revealed that the EF instrument surfaces were the smoothest in comparison to other files investigated, while the files were the most prone to the fractures and demonstrated the longest lifespan during the cyclic fatigue test [14]. The investigation of new CM and EDM instruments has shown the typical and previously identified superficial morphological features—milling and scratching marks on the CM and crater-like surface microstructure for EDM instruments, indicating the electro-discharge machining nature of the manufacturing process [13]. The SEM assessment of instruments subjected to cyclic fatigue test revealed a substantial number of defects and flute integrity changes on EF instruments, while these defects were very uncommon on CM files. Meanwhile, no visible defects or changes in the integrity of the flutes or tip were detected on EDM instruments. On the cross-sectional micrographs, the microcracks at the cutting edges as a sign of the crack origin as well as typical initiation and propagation zones were identified on all files tested. However, on EDM instruments the dimpled area was more centered, while for EF and CM instruments more eccentric and wider, indicating the slightly different nature of the fractures. These findings are in agreement with previous reports, demonstrating the different fracture mechanism depending on the thermal treatment method of the NiTi alloy [13,40].

## 5. Conclusions

Within the limitations of this ex vivo study, it can be concluded that CM, EDM and EF thermally treated rotary NiTi endodontic instruments were equally effective in shaping and centering abilities and instrumented curved root canals with no considerable shaping mishaps and errors. However, the ED instruments, made from the Fire-Wire™ NiTi alloy demonstrated significantly higher resistance to cyclic fatigue in comparison to EDM and CM files. The manufacturing process of electrical discharge machining improves the resistance to fracture of CM alloy-made instruments, increasing the fatigue lifetime of EDM prototypes.

## Figures and Tables

**Table 1:** Three-dimensional (3D) morphometric parameters between three file systems evaluated (mean values ± SD).

Group	*n*	Volume of Untreated Canal (mm^3^)	Volume of Removed Dentin (mm^3^)	Percentage of Unprepared Canal Surface
CM	20	3.44 ± 1.10	2.14 ± 0.49	41.16 ± 4.28
EDM	20	3.48 ± 1.12	2.12 ± 0.51	42.06 ± 4.11
EF	20	3.42 ± 1.04	2.04 ± 0.53	43.28 ± 4.19

**Table 2:** Root canal transportation after use of the thermally-treated instrument systems (mm ± SD).

Group	*n*	Coronal Third	Middle Third	Apical Third
CM	20	0.05 ± 0.02	0.06 ± 0.01	0.07 ± 0.02
EDM	20	0.04 ± 0.01	0.06 ± 0.01	0.06 ± 0.01
EF	20	0.05 ± 0.01	0.07 ± 0.02	0.07 ± 0.01

**Table 3:** The range of rotation time, mean ± standard deviation of the number of cycles to fracture (NCF) and length of fractured fragment between three thermally-treated NiTi file systems.

	*n*	CM	EDM	EF
The range of rotation time (s)	20	76–244 *	300–491 *	421–1164 *
NCF	20	1143 ± 472 *	3132 ± 481 *	4504 ± 1428 *
Length of the fragment (mm)	20	4.21 ± 0.27	4.15 ± 0.18	4.12 ± 0.42

* Indicate significant statistically significant differences between groups (*p* < 0.05).

## References

[1] Peters O.A. (2004). Current challenges and concepts in the preparation of root canal systems: A review. J. Endod..

[2] Gutmann J.L., Gao Y. (2012). Alteration in the inherent metallic and surface properties of nickel-titanium root canal instruments to enhance performance, durability and safety: A focused review. Int. Endod. J..

[3] Schäfer E., Bürklein S. (2012). Impact of nickel-titanium instrumentation of the root canal on clinical outcomes: A focused review. Odontology.

[4] Vaudt J., Bitter K., Neumann K., Kielbassa A.M. (2009). Ex vivo study on root canal instrumentation of two rotary nickel-titanium systems in comparison to stainless steel hand instruments. Int. Endod. J..

[5] Andreasen J.O., Farik B., Munksgaard E.C. (2002). Long-term calcium hydroxide as a root canal dressing may increase risk of root fracture. Dent. Traumatol..

[6] Pedullà E., Lo Savio F., Boninelli S., Plotino G., Grande N.M., La Rosa G., Rapisarda E. (2016). Torsional and Cyclic Fatigue Resistance of a New Nickel-Titanium Instrument Manufactured by Electrical Discharge Machining. J. Endod..

[7] Silva E.J.N.L., Martins J.N.R., Lima C.O., Vieira V.T.L., Braz Fernandes F.M., De-Deus G., Versiani M.A. (2020). Mechanical Tests, Metallurgical Characterization, and Shaping Ability of Nickel-Titanium Rotary Instruments: A Multimethod Research. J. Endod..

[8] Silva E.J.N.L., Giraldes J.F.N., de Lima C.O., Vieira V.T.L., Elias C.N., Antunes H.S. (2019). Influence of heat treatment on torsional resistance and surface roughness of nickel-titanium instruments. Int. Endod. J..

[9] Zupanc J., Vahdat-Pajouh N., Schäfer E. (2018). New thermomechanically treated NiTi alloys—A review. Int. Endod. J..

[10] Kaval M.E., Capar I.D., Ertas H. (2016). Evaluation of the Cyclic Fatigue and Torsional Resistance of Novel Nickel-Titanium Rotary Files with Various Alloy Properties. J. Endod..

[11] Zhao D., Shen Y., Peng B., Haapasalo M. (2013). Micro-computed tomography evaluation of the preparation of mesiobuccal root canals in maxillary first molars with Hyflex CM, twisted files, and K3 instruments. J. Endod..

[12] Topçuoǧlu H.S., Topçuoǧlu G., Akti A., Düzgün S. (2016). In Vitro Comparison of Cyclic Fatigue Resistance of ProTaper Next, HyFlex CM, OneShape, and ProTaper Universal Instruments in a Canal with a Double Curvature. J. Endod..

[13] Pirani C., Iacono F., Generali L., Sassatelli P., Nucci C., Lusvarghi L., Gandolfi M.G., Prati C. (2016). HyFlex EDM: Superficial features, metallurgical analysis and fatigue resistance of innovative electro discharge machined NiTi rotary instruments. Int. Endod. J..

[14] Bueno C.R.E., Cury M.T.S., Vasques A.M.V., Sivieri-Araújo G., Jacinto R.C., Gomes-Filho J.E., Cintra L.T.A., Dezan E. (2019). Cyclic fatigue resistance of novel Genius and Edgefile nickel-titanium reciprocating instruments. Braz. Oral Res..

[15] Yılmaz F., Eren İ., Eren H., Badi M.A., Ocak M., Çelik H.H. (2020). Evaluation of the Amount of Root Canal Dentin Removed and Apical Transportation Occurrence after Instrumentation with ProTaper Next, OneShape, and EdgeFile Rotary Systems. J. Endod..

[16] Razcha C., Zacharopoulos A., Anestis D., Mikrogeorgis G., Zacharakis G., Lyroudia K. (2020). Micro-Computed Tomographic Evaluation of Canal Transportation and Centering Ability of 4 Heat-Treated Nickel-Titanium Systems. J. Endod..

[17] Schneider S.W. (1971). A comparison of canal preparations in straight and curved root canals. Oral Surg. Oral Med. Oral Pathol..

[18] Del Rio C.E. (1996). Comparison of nickel-titanium and stainless steel hand-file instrumentation using computed tomography. J. Endod..

[19] Drukteinis S., Peciuliene V., Bendinskaite R., Brukiene V., Maneliene R., Nedzinskiene E. (2020). Shaping Ability, Cyclic Fatigue Resistance and Fractographic Analysis of Hybrid and Reciprocating Nickel Titanium Endodontic Instruments. Metals.

[20] Villas-Bôas M.H., Bernardineli N., Cavenago B.C., Marciano M., Del Carpio-Perochena A., De Moraes I.G., Duarte M.H., Bramante C.M., Ordinola-Zapata R. (2011). Micro-computed tomography study of the internal anatomy of mesial root canals of mandibular molars. J. Endod..

[21] Pinheiro S.R., Alcalde M.P., Vivacqua-Gomes N., Bramante C.M., Vivan R.R., Duarte M.A.H., Vasconcelos B.C. (2018). Evaluation of apical transportation and centring ability of five thermally treated NiTi rotary systems. Int. Endod. J..

[22] De-Deus G., Canabarro A., Alves G.G., Marins J.R., Linhares A.B.R., Granjeiro J.M. (2012). Cytocompatibility of the ready-to-use bioceramic putty repair cement iRoot BP Plus with primary human osteoblasts. Int. Endod. J..

[23] Saber S.E.D.M., Nagy M.M., Schäfer E. (2015). Comparative evaluation of the shaping ability of ProTaper Next, iRaCe and Hyflex CM rotary NiTi files in severely curved root canals. Int. Endod. J..

[24] Donnermeyer D., Viedenz A., Schäfer E., Bürklein S. (2020). Impact of new cross-sectional designs on the shaping ability of rotary NiTi instruments in S-shaped canals. Odontology.

[25] Shen Y., Peng B., Yang Y., Ma J., Haapasalo M. (2015). What do different tests tell about the mechanical and biological properties of bioceramic materials?. Endod. Top..

[26] Venino P.M., Citterio C.L., Pellegatta A., Ciccarelli M., Maddalone M. (2017). A Micro–computed Tomography Evaluation of the Shaping Ability of Two Nickel-titanium Instruments, HyFlex EDM and ProTaper Next. J. Endod..

[27] Versiani M.A., Carvalho K.K.T., Mazzi-Chaves J.F., Sousa-Neto M.D. (2018). Micro–computed Tomographic Evaluation of the Shaping Ability of XP-endo Shaper, iRaCe, and EdgeFile Systems in Long Oval-shaped Canals. J. Endod..

[28] Paqué F., Ganahl D., Peters O.A. (2009). Effects of Root Canal Preparation on Apical Geometry Assessed by Micro-Computed Tomography. J. Endod..

[29] Fernandes P.O.F., Freire L.G., Iglecias E.F., Vieira B.R., Zuolo M.L., Gavini G. (2020). Assessment of Mechanical Root Canal Preparation with Centric Reciprocating or Eccentric Rotary Kinematics: A Micro–computed Tomographic Study. J. Endod..

[30] Carvalho A.P.L., Nardello L.C.L., Fernandes F.S., Bruno F.P., Paz L.R., Iglecias E.F., Honório H.M., Mayer M.P.A., Gavini G., Pinheiro E.T. (2020). Effects of Contemporary Irrigant Activation Schemes and Subsequent Placement of an Interim Dressing on Bacterial Presence and Activity in Root Canals Associated with Asymptomatic Apical Periodontitis. J. Clin. Med..

[31] Neves M.A.S., Provenzano J.C., Rôças I.N., Siqueira J.F. (2016). Clinical Antibacterial Effectiveness of Root Canal Preparation with Reciprocating Single-instrument or Continuously Rotating Multi-instrument Systems. J. Endod..

[32] Wu M.K., R’oris A., Barkis D., Wesselink P.R. (2000). Prevalence and extent of long oval canals in the apical third. Oral Surg. Oral Med. Oral Pathol. Oral Radiol. Endod..

[33] Bürklein S., Schäfer E. (2013). Critical evaluation of root canal transportation by instrumentation. Endod. Top..

[34] Plotino G., Testarelli L., Al-Sudani D., Pongione G., Grande N.M., Gambarini G. (2014). Fatigue resistance of rotary instruments manufactured using different nickel-titanium alloys: A comparative study. Odontology.

[35] Hülsmann M., Donnermeyer D., Schäfer E. (2019). A critical appraisal of studies on cyclic fatigue resistance of engine-driven endodontic instruments. Int. Endod. J..

[36] Topçuoğlu H.S., Topçuoğlu G., Kafdağ Ö., Balkaya H. (2020). Effect of two different temperatures on resistance to cyclic fatigue of one Curve, EdgeFile, HyFlex CM and ProTaper next files. Aust. Endod. J..

[37] Dosanjh A., Paurazas S., Askar M. (2017). The Effect of Temperature on Cyclic Fatigue of Nickel-titanium Rotary Endodontic Instruments. J. Endod..

[38] Adıgüzel M., Öztekin F. (2020). Comparison of the resistance to cyclic fatigue of One Curve, One Shape, 2Shape and EdgeFile X3 files in simulated single and S-shaped (double) curvatures. Int. Dent. Res..

[39] Goo H.J., Kwak S.W., Ha J.H., Pedullà E., Kim H.C. (2017). Mechanical Properties of Various Heat-treated Nickel-titanium Rotary Instruments. J. Endod..

[40] Iacono F., Pirani C., Generali L., Bolelli G., Sassatelli P., Lusvarghi L., Gandolfi M.G., Giorgini L., Prati C. (2017). Structural analysis of HyFlex EDM instruments. Int. Endod. J..

[41] Pedullà E., Plotino G., Grande N.M., Scibilia M., Pappalardo A., Malagnino V.A., Rapisarda E. (2014). Influence of rotational speed on the cyclic fatigue of Mtwo instruments. Int. Endod. J..

